# A Population-Based Evaluation of Polymicrobial *Staphylococcus aureus* Bacteremia

**DOI:** 10.3390/pathogens11121499

**Published:** 2022-12-08

**Authors:** Joya-Rita Hindy, Juan A. Quintero-Martinez, Brian D. Lahr, Daniel C. DeSimone, Larry M. Baddour

**Affiliations:** 1 Division of Public Health, Infectious Diseases and Occupational Health, Department of Medicine, College of Medicine, Mayo Clinic, Rochester, MN 55905, USA; 2 Division of Clinical Trials & Biostatistics, Department of Quantitative Health Sciences, College of Medicine, Mayo Clinic, Rochester, MN 55905, USA; 3 Department of Cardiovascular Disease, College of Medicine, Mayo Clinic, Rochester, MN 55905, USA

**Keywords:** *Staphylococcus aureus*, bacteremia, polymicrobial, population-based, mortality, incidence

## Abstract

Objective: To provide an evaluation of incidence and six-month mortality rates of polymicrobial *Staphylococcus aureus* bacteremia (p-SAB) in the United States (US). Methods: A retrospective population-based study of all incident adults with monomicrobial SAB (m-SAB) and p-SAB in Olmsted County, Minnesota (MN) from 1 January 2006, through 31 December 2020, was conducted. Demographics, clinical characteristics, in-hospital outcomes, and six-month survival were compared between groups. Results: Overall, 31 incident p-SAB cases occurred during the 15-year study period, corresponding to an overall age- and sex-standardized incidence rate of 1.9/100,000 person-years (95% CI, 1.3–2.6). One-third of p-SAB cases were due to MRSA, and almost one-half (15/31) were caused by Gram-positive bacteria. As compared to the 541 cases with incident m-SAB, p-SAB patients were more likely to have a catheter-related infection (*p* = 0.008) and less likely to be community-acquired cases (*p* = 0.027). The unadjusted risk of six-month mortality was greater in the p-SAB group (14/31, 45.2%) compared to the m-SAB group (144/541, 26.6%) (HR = 1.94, 95% CI = 1.12–3.36, *p* = 0.018). After adjusting for relevant covariates, this difference approached significance (HR = 1.93, 95% = CI 0.96–3.87, *p* = 0.064). Conclusions: To our knowledge, the current investigation represents the only US population-based study evaluating p-SAB patients. We found lower incidence rates for p-SAB than previously reported, with almost one-half of the cases caused by Gram-positive bacteria. Furthermore, these patients had poor survival compared to incident m-SAB cases.

## 1. Introduction

Polymicrobial bacteremia, representing 5–20% of all bacteremia cases, often originates from intraabdominal infections, complicated urinary tract infections, or infected intravascular catheters, and is usually associated with worse outcomes [1,2,3]. Although *Staphylococcus aureus* is the second most common cause of bacteremia and leads to one of the highest mortality rates among all pathogens causing bacteremias [4,5,6,7], polymicrobial *S. aureus* bacteremia (p-SAB) has been infrequently reported. Few studies have focused on p-SAB over the past decade [8,9,10], including population-based investigations [11,12].

Therefore, the aim of our study was to compare monomicrobial SAB (m-SAB) patients identified in a prior investigation [13] with p-SAB cases based on baseline clinical characteristics and outcomes in a population-based cohort. We also further investigated whether p-SAB was independently associated with six-month mortality.

## 2. Material and Methods

A large, unified database entitled the “Rochester Epidemiological Project” (REP) was developed in Olmsted County, Minnesota (MN) in 1966 and was used in this investigation; every resident of the county has a unique medical file regardless of provider and whether residents present in an inpatient, outpatient, or emergent setting [14]. This study was approved by the Institutional Review Boards (IRB) at both Mayo Clinic (MC) (IRB#: 20-012295) and Olmsted Medical Center (OMC) (IRB#: 061-OMC-20), the only two facilities having clinical microbiology laboratories in Olmsted County. From 1 January 2006, through 31 December 2020, and after excluding 45 patients who denied research authorization by opt-out choice, 541 adult patients with an initial episode of m-SAB and 31 adult patients with p-SAB were identified by the REP browser. Four patients had both monomicrobial and polymicrobial but were a few years apart and were included in both groups.

Electronic health records (EHR) of patients with either m-SAB or p-SAB were reviewed. The Charlson Comorbidity Index (CCI) was calculated [15], and data on the site of onset and complicated SAB were collected. The source of SAB and metastatic infections were defined according to history, physical examination, and laboratory and imaging findings retrieved from a patient’s EHR. Recovery of *S. aureus* by culture from a potential source of infection was not required. Six-month mortality was the primary outcome. Secondary outcomes included the incidence rate of p-SAB, hospital length of stay, in-hospital mortality, SAB complications, and re-infection as defined by having a positive blood culture detected within six months of negative follow-up blood cultures [16].

If a patient resided in a long-term care facility or a nursing home, was admitted to an acute care hospital for 48 h or more within 90 days prior to SAB, or underwent hemodialysis, intravenous therapy, chemotherapy, wound care, or specialized nursing care within 30 days prior to SAB, SAB was defined as healthcare-associated infection [17]. If a patient had a positive blood culture obtained at admission to the hospital or within 48 h after admission and did not fulfill the criteria for healthcare-associated SAB, it was defined as community-acquired [17]. If a patient had a positive blood culture obtained after two days of hospital admission, SAB was characterized as nosocomial. If patients were transferred from other medical centers, the duration of hospitalization was calculated from the first admission date [17]. If patients had indwelling devices, persistent fever, or positive blood cultures within two to four days after anti-biotherapy initiation and control of any infection focus, signs of metastatic infection, or findings of infective endocarditis (IE) on echocardiography, SAB was characterized as complicated [18].

When another organism was isolated simultaneously with *S. aureus*, SAB was defined as polymicrobial. However, if the other isolate was a usual contaminant (such as *Corynebacterium* spp., *Bacillus* spp., or coagulase-negative *Staphylococcus*), SAB was considered monomicrobial unless these organisms were recovered in two or more blood cultures [19].

## 3. Statistical Analysis

Incidence rates of p-SAB were calculated for men and women in age categories by dividing the number of cases by person-time of follow-up, expressed per 100,000 person-years. Sex-specific incidence rates across all ages were standardized to the age distribution of the US white population in 2010, and an overall incidence rate was standardized to the age and sex distribution of the same background population. Ninety-five percent confidence intervals (CIs) for rates were calculated assuming a Poisson distribution.

Baseline differences between the p-SAB and m-SAB groups were assessed using simple bivariate comparisons (i.e., Wilcoxon rank sum, Pearson χ^2^, or Fisher exact tests). For outcomes analysis, hospital length of stay, six-month mortality, and re-infection were each analyzed as censored time-to-event outcomes. Survival up to six months was analyzed by Kaplan-Meier and Cox proportional hazards methods. Univariable and multivariable Cox regression models were used to quantify the risk of death according to the p-SAB group versus the m-SAB group, before and after adjusting for prespecified covariates: age, sex, diabetes, liver disease, chronic kidney disease, CCI, unknown source of SAB, site of SAB acquisition, MRSA, ICU admission and ID consult. Results are reported as hazard ratios (HR) and 95% CI. In analyses taking the competing risk of death into account, time to hospital discharge and time to re-infection were estimated by the cumulative incidence function and compared according to Gray’s test. All analyses were conducted using the R statistical package (version 4.0.3; R Foundation, Vienna, Austria).

## 4. Results

### 4.1. Incidence of p-SAB

Overall, 31 patients with incident p-SAB were identified in Olmsted County, MN from 2006 to 2020. The age-standardized incidence rate for males was 2.6/100,000 person-years (95% CI, 1.4–3.8), compared with 1.5/100,000 person-years (95% CI, 0.7–2.3) for females. The age- and sex-standardized incidence rate for p-SAB was 1.9/100,000 person-years (95% CI, 1.3–2.6). The incidence of p-SAB increased with age (Table 1). P-SAB cases were 5.5% of all SAB cases.

### 4.2. Microbiology of p-SAB

**Figure 1:**
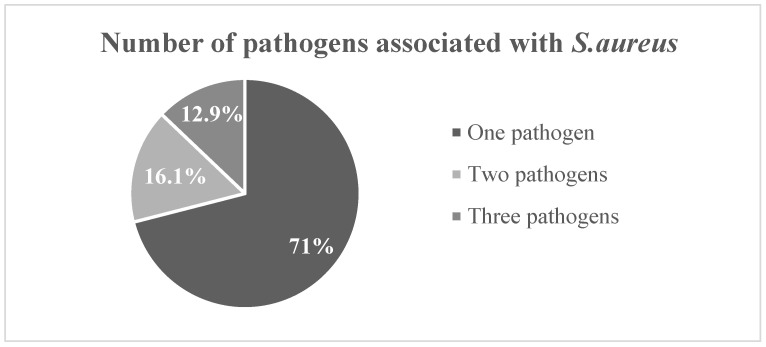

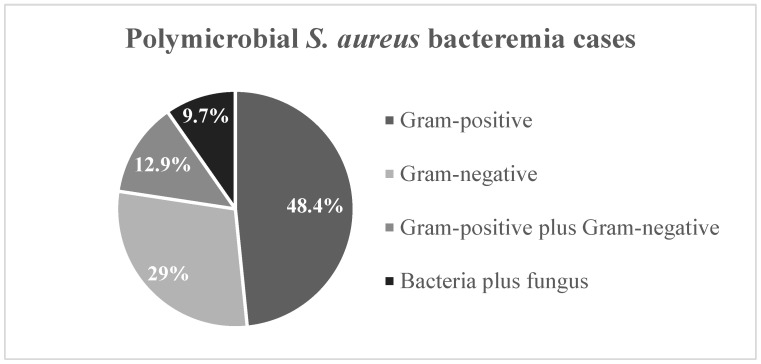
Microbiology of polymicrobial *S. aureus* bacteremia.

Nearly half of the p-SAB cases (15/31, 48.2%) were caused by Gram-positive bacteria (Figure 1). In addition to *S. aureus*, most p-SAB cultures (22/31, 71.0%) grew one organism and the highest number of pathogens detected in other culture sets was three (Table 2).

### 4.3. Baseline Characteristics of p-SAB and m-SAB

The demographic and clinical characteristics of the 31 incident p-SAB cases were compared to 541 incident m-SAB cases identified during the same 15-year period (Table 3). Patients with p-SAB had similar age and sex distributions to those of m-SAB cases, and the majority of baseline characteristics of p-SAB patients were not significantly different. Site of SAB acquisition, however, differed between groups (*p* = 0.027), with the p-SAB group (vs. m-SAB) representing more healthcare-associated cases (67.7% vs. 49.2%) and fewer community-acquired cases (16.1% vs. 40.3%). The most common sources of p-SAB were catheter-related bloodstream infections (CRBSI) (9/31, 29.0%) and skin and soft tissue infections (9/31, 29.0%), followed by catheter-associated urinary tract infections (CAUTI) (7/31, 22.6%) and unknown source (7/31, 22.6%). Compared with the m-SAB group, a higher proportion of p-SAB cases were due to CRBSI or CAUTI (both *p* < 0.01).

### 4.4. Outcomes in p-SAB and m-SAB Patients

**Figure 2:**
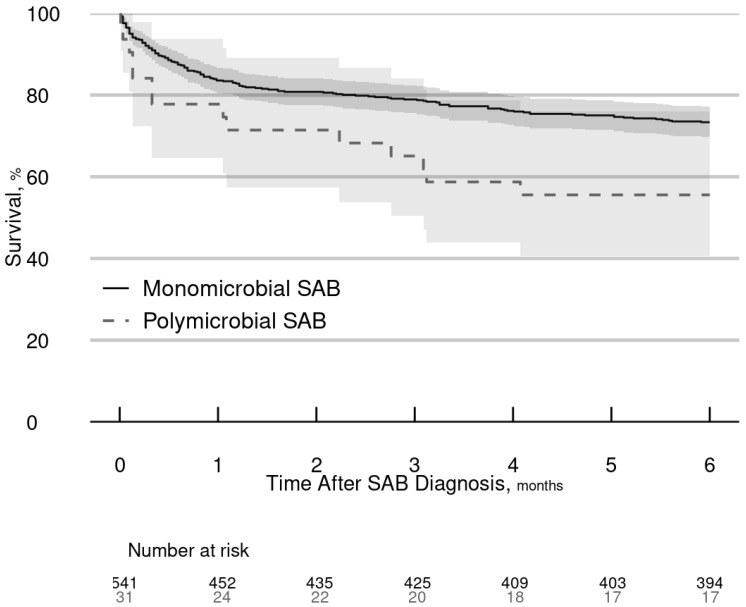
Survival rates of SAB patients over six months. Abbreviations: SAB, *Staphylococcus aureus* bacteremia.

There were no significant differences between the p-SAB and m-SAB groups for hospital length of stay, complications, or in-hospital mortality (Table 4). During the six-month follow-up period, 26.7% (144/541) of patients in the m-SAB group died compared with 45.2% (14/31) of patients in the p-SAB group (Figure 2). In unadjusted analysis, p-SAB (vs. m-SAB) was associated with an increased risk of six-month mortality (HR = 1.94, 95% CI = 1.12–3.36, *p* = 0.018). After adjusting for relevant covariates (age, sex, diabetes mellitus, liver disease, chronic kidney disease, Charlson Comorbidity Index, unknown source of SAB, site of SAB acquisition, MRSA, ICU admission, and ID consult), the association of p-SAB with six-month mortality was similar in magnitude but only marginally significant (HR = 1.93, 95% = CI 0.96–3.87, *p* = 0.064). Regarding covariates, older age, female sex, unknown source of SAB, and ICU admission were associated with an elevated risk of dying within six months; in contrast, ID consult was associated with a reduced risk of dying but only within the first two weeks of diagnosis (Table 5).

## 5. Discussion

To our knowledge, the current investigation represents the only US population-based study evaluating p-SAB patients. During the 15-year study period, we report an incidence rate for p-SAB that is one of the lowest described to date. Nearly half of p-SAB cases were caused by Gram-positive bacteria. Furthermore, these patients experienced marginally worse six-month survival as compared to that of m-SAB cases.

As a clinical entity, p-SAB has not been well-characterized. Moreover, in a systematic review of 26 population-based investigations of SAB incidence trends, only 2 studies addressed p-SAB and the majority did not specify whether SAB was monomicrobial or polymicrobial [20]. One population-based study conducted in Iceland reported p-SAB in 5.1%, 8.0%, and 5.3% of SAB cases during the respective periods of 1995–1999, 2000–2004, and 2005–2008 [12]. The second population-based investigation conducted in Victoria, Canada during 1998–2005 described a percentage of cases due to p-SAB of 8% [11]. Other single-center investigations described various percentages of p-SAB: 14% in Southwestern Ontario, Canada [21], 6.1% in Detroit, Michigan [8], 10% in Seoul, South Korea [9], 15.5% in Hangzhou, China [10], and 18% in Karachi, Pakistan [22]. The Chinese study reported higher in-hospital, 7-day, 14-day, and 28-day mortality rates in patients with p-SAB as compared to that in patients with m-SAB [10]. The Korean investigation demonstrated p-SAB as an independent risk factor associated with mortality [9]. Most of the p-SAB patients in these above-mentioned studies were males and their ages ranged from 51 to 70 years [8,10,11]. The most common source of p-SAB in the other US study was endovascular, which is comparable to our findings [8], though it reported a higher prevalence of both diabetes mellitus and hemodialysis use [8]. However, both US-based investigations noted a higher prevalence of diabetes mellitus and CKD as compared to that in the Chinese study [10].

Similarly to our findings, the Korean single-center study reported that Gram-positive pathogens were more commonly identified in p-SAB, and *Enterococcus* spp. was most frequently identified [9]. However, the majority of co-pathogens detected in p-SAB were Gram-negative bacilli in the US and Chinese single-center studies [8,10]. A case-control study reported that patients with MRSA more often had polymicrobial bacteremia, usually with other Gram-positive organisms, and were more frequently culture positive at other sites when compared to that of MSSA [21]. Several risk factors associated with p-SAB have been reported, including older age, presence of biliary tract catheters, intra-abdominal, respiratory or urinary source of infection, burn injury, neutropenia, need of blood transfusion, use of mechanical ventilation, and length of hospital stay prior to the onset of p-SAB [9,10,11]. In addition, patients with p-SAB had worse outcomes (including seven-day and bacteremia-related mortality rates) compared with that of m-SAB [9].

The benefits of ID consultation in the setting of SAB have led to the increased ordering of follow-up blood cultures, prompt request for echocardiography, early recognition and control of metastatic foci of infection, early antibiotic selection and dosing with an appropriate duration of therapy, and a shorter hospital stay, reducing the rate of SAB relapse and insuring a prompt diagnosis of IE [23,24,25]. Since April 2016 at Mayo Clinic, the microbiology laboratory reporting of SAB has included a prompt to order ID consultation. As a result, in part, 79.2% of our total cohort (90.3% of p-SAB cases) had ID consultation as compared to only 63% of SAB patients in one of the largest Canadian multicenter retrospective studies [26].

The current study has several limitations. First, the number of patients with p-SAB was small, limiting the statistical power of our bivariate comparisons and precluding a multivariable analysis to assess risk factors associated with p-SAB. Second, our population-based study was conducted in Olmsted County, MN was comprised primarily of non-Hispanic whites and thus our findings may not be generalizable to diverse multi-ethnic populations. However, a major strength of this study is that it was based on a population-based investigation, rather than a single-center experience which is susceptible to referral bias and incomplete patient follow-up.

## 6. Conclusions

To our knowledge, the current investigation represents the only US population-based study evaluating p-SAB patients. In this population studied over a 15-year period, we found lower incidence rates for p-SAB than previously reported, with almost one-half of these cases caused by Gram-positive bacteria. Compared with m-SAB cases, p-SAB patients had a marginally increased risk of dying within six months of diagnosis.

## Figures and Tables

**Table 1:** Incidence rates of polymicrobial SAB by age category, calendar year, and sex.

	Female		Male		Total	
	No.	IR	No.	IR	No.	IR
**Age Category**						
18–49	2	0.4	2	0.4	4	0.4
50–64	5	2.3	6	3.0	11	2.6
65–79	4	3.3	7	6.9	11	5.0
80–100	2	3.8	3	9.1	5	5.9
All ages	13	1.4	18	2.2	31	1.8
**Calendar Year**						
2006–2010	6	2.1	4	1.6	10	1.8
2011–2015	3	1.0	2	0.7	5	0.9
2016–2020	4	1.2	12	4.2	16	2.6
**Overall**	13	1.5 (0.7–2.3) ^1^	18	2.6 (1.4–3.8) ^1^	31	1.9 (1.3–2.6) ^2^

No., number of incident SAB cases; IR, incidence rate (number of incident cases per 100,000 person-years), ^1^ Age-adjusted incidence rate (95% confidence interval), ^2^ Age- and sex-adjusted incidence rate (95% confidence interval), Abbreviations: IR, incidence rate; SAB, *S. aureus* bacteremia.

**Table 2:** Microbiology of polymicrobial SAB.

Cases of Polymicrobial SAB	*n* = 31
** Gram positive**	15 (48.4%)
*Bacillus cereus/thuringiensis*	1 (3.2%)
*Enterococcus species*	3 (9.7%)
*Enterococcus faecalis*	3 (9.7%)
*Enterococcus faecalis + Staphylococcus warneri + Staphylococcus epidermidis*	1 (3.2%)
*Enterococcus faecium*	1 (3.2%)
*Staphylococcus epidermidis*	1 (3.2%)
*Streptococcus agalactiae*	2 (6.5%)
*Viridans group streptococci*	3 (9.7%)
**Gram negative**	9 (29%)
*Acinetobacter species*	1 (3.2%)
*Escherichia coli*	2 (6.5%)
*Klebsiella oxytoca*	1 (3.2%)
*Proteus mirabilis*	1 (3.2%)
*Pseudomonas aeruginosa*	2 (6.5%)
*Pseudomonas aeruginosa + Enterobacter cloacae*	1 (3.2%)
*Unidentified Gram-negative bacillus anaerobic*	1 (3.2%)
**Gram positive plus Gram negative**	4 (12.9%)
*Enterococcus faecalis + Enterobacter cloacae*	1 (3.2%)
*Escherichia coli + Streptococcus mitis group + Gemella haemolysans*	1 (3.2%)
*Klebsiella pneumoniae + Enterococcus faecalis + Veillonella species*	1 (3.2%)
*Pseudomonas aeruginosa + Enterococcus faecalis*	1 (3.2%)
**Bacteria plus Fungus**	3 (9.7%)
*Proteus mirabilis + Escherichia coli + Candida glabrata*	1 (3.2%)
*Rothia + Candida albicans*	1 (3.2%)
*Staphylococcus devriesei/haemolyticus + Candida parapsilosis*	1 (3.2%)

Abbreviation: SAB, *S. aureus* bacteremia.

**Table 3:** Baseline characteristics of SAB cases (m-SAB VS p-SAB).

Characteristic	*n*	Monomicrobial SAB (*n* = 541)	Polymicrobial SAB (*n* = 31)	*p*-Value
Age, years	572	66.8 (54.4–78.5)	65.5 (54.9–77.7)	0.877 ^1^
Sex: Female	572	214 (39.6%)	13 (41.9%)	0.792 ^2^
Diabetes Mellitus	572	249 (46.0%)	9 (29.0%)	0.064 ^2^
Liver Disease	572	156 (28.8%)	14 (45.2%)	0.053 ^2^
Chronic Kidney Disease	572			0.789 ^2^
No		322 (59.5%)	18 (58.1%)	
Yes, without HD		168 (31.1%)	11 (35.5%)	
Yes, with HD		51 (9.4%)	2 (6.5%)	
Charlson Comorbidity Index	572	6.0 (3.0–9.0)	8.0 (2.5–10.5)	0.265 ^1^
Source of SAB	572			
SSTI		203 (37.5%)	9 (29.0%)	0.341 ^2^
CRBSI		67 (12.4%)	9 (29.0%)	0.008 ^2^
CAUTI		43 (7.9%)	7 (22.6%)	0.005 ^2^
Unknown		108 (20.0%)	7 (22.6%)	0.724 ^2^
Pneumonia		70 (12.9%)	1 (3.2%)	0.111 ^2^
Septic Arthritis		52 (9.6%)	1 (3.2%)	0.233 ^2^
Other		86 (15.9%)	0 (0.0%)	0.016 ^2^
Site of Infection Onset	570			0.027 ^2^
Nosocomial		57 (10.6%)	5 (16.1%)	
Healthcare Associated		265 (49.2%)	21 (67.7%)	
Community Acquired		217 (40.3%)	5 (16.1%)	
Type of SAB: MRSA	560	232 (43.8%)	10 (33.3%)	0.261 ^2^
ICU Admission	571	144 (26.7%)	9 (29.0%)	0.772 ^2^
ID Consult	571	424 (78.5%)	28 (90.3%)	0.116 ^2^
Hospitalization	572	532 (98.3%)	31 (100.0%)	1.000 ^3^
TTE Obtained	569	178 (33.1%)	16 (51.6%)	0.034 ^2^
TEE Obtained	567	257 (47.9%)	7 (22.6%)	0.006 ^2^

Values represent median (quartile 1 to quartile 3) for continuous variables and frequency (percentage) for categorical variables. *n* is the number of non-missing values. *p* values are by ^1^ Wilcoxon rank sum, ^2^ Pearson χ^2^, or ^3^ Fisher exact tests. Abbreviations: CAUTI, catheter-associated urinary tract infection; CRBSI, catheter-related bloodstream infections; HD, hemodialysis; MRSA, ICU, intensive care unit; ID, infectious diseases; methicillin-resistant *Staphylococcus aureus*; MSSA, methicillin-susceptible *Staphylococcus aureus*; SAB, *Staphylococcus aureus* bacteremia, TTE, transthoracic echocardiography; TEE, transesophageal echocardiography.

**Table 4:** Complications and outcomes of SAB patients.

	*n*	Monomicrobial SAB (*n* = 541)	Polymicrobial SAB (*n* = 31)	*p* Value
**Complication**				
Complicated Bacteremia	571	207 (38.3%)	9 (29.0%)	0.299 ^1^
Infective Endocarditis	569	39 (7.2%)	2 (6.5%)	0.867 ^1^
Vertebral Osteomyelitis	571	30 (5.6%)	1 (3.2%)	0.578 ^1^
Non-vertebral Osteomyelitis	571	40 (7.4%)	4 (12.9%)	0.264 ^1^
Septic Arthritis	570	47 (8.7%)	1 (3.2%)	0.284 ^1^
Psoas Abscess	571	4 (0.7%)	0 (0.0%)	1.000 ^2^
Splenic Abscess	571	3 (0.6%)	0 (0.0%)	1.000 ^2^
Renal Abscess	571	2 (0.4%)	0 (0.0%)	1.000 ^2^
Deep Seated Abscess	571	49 (9.1%)	0 (0.0%)	0.079 ^1^
Pneumonitis	571	30 (5.6%)	0 (0.0%)	0.178 ^1^
Cerebral Abscess	571	5 (0.9%)	0 (0.0%)	1.000 ^2^
Meningitis	571	2 (0.4%)	0 (0.0%)	1.000 ^2^
Stroke	571	4 (0.7%)	1 (3.2%)	0.244 ^2^
Septic Emboli	571	16 (3.0%)	2 (6.5%)	0.255 ^2^
Epidural Abscess	571	9 (1.7%)	0 (0.0%)	1.000 ^2^
Altered Mental Status	571	11 (2.0%)	0 (0.0%)	1.000 ^2^
**Outcome**				
In-hospital Mortality	559	59 (11.2%)	6 (19.4%)	0.167 ^1^
Hospital Length of Stay, days	559	10 (6–19)	12 (7–37)	0.254 ^3^
6-month Mortality	572	144 (26.7%)	14 (45.2%)	0.018 ^4^
6-month Reinfection	572	7 (1.3%)	0 (0.0%)	0.524 ^3^

Binary outcome variables are reported as frequency (percentage); time-to-event outcomes are presented with cumulative incidence or quartile estimates based on the cumulative incidence function accounting for the competing risk of death. *n* is the number of non-missing values for each outcome. *p* values are by ^1^ Pearson χ^2^ test, ^2^ Fisher exact test, ^3^ Gray test, or ^4^ Cox (unadjusted) regression analysis. Abbreviation: SAB, *S. aureus* bacteremia.

**Table 5:** Determinants of six-month mortality in SAB patients.

Predictor	Comparison	HR (95% CI)	*p*
SAB group	Polymicrobial vs. Monomicrobial	1.93 (0.96–3.87)	0.064
Age	78.5 years vs. 54.4 years	3.15 (2.33–4.27)	<0.001
Sex	Female vs. Male	1.39 (1.01–1.91)	0.044
Diabetes mellitus	Yes vs. No	0.75 (0.52–1.07)	0.115
Liver disease	Yes vs. No	1.06 (0.71–1.58)	0.778
Chronic kidney disease			0.231
	Yes, without HD vs. No	1.37 (0.92–2.03)	
	Yes, with HD vs. No	1.59 (0.78–3.26)	
Charlson Comorbidity Index	10 vs. 3	1.27 (0.89–1.80)	0.301
Source of SAB	Unknown vs. Known	1.75 (1.22–2.51)	0.002
Site of infection onset			0.492
	HA vs. Nosocomial	0.77 (0.47–1.26)	
	CA vs. Nosocomial	0.73 (0.43–1.24)	
Type of SAB	MRSA vs. MSSA	0.86 (0.62–1.21)	0.392
ICU admission	Yes vs. No	2.23 (1.59–3.13)	<0.001
ID consult	Yes vs. No; phase = early ^1^	0.31 (0.19–0.50)	<0.001
	Yes vs. No; phase = late ^1^	0.94 (0.52–1.70)	0.827

Results are from a multivariable Cox regression model for predicting time to six-month mortality. Continuous variables were modeled with 3-knot restricted cubic splines to allow for nonlinear associations, with HRs calculated per IQR increase (comparing 75th and 25th percentile values). ^1^ Evidence of non-proportional hazards (*p* = 0.004) indicated a differential effect of ID consult over the six-month follow-up period. To reflect this difference, two distinct phases were chosen, one representing early survival (0–14 days) and the second representing late survival (15 days to six months), with each yielding separate estimates of the effect of ID consult. Abbreviations: CA, community-acquired; HA, healthcare-associated; HD, hemodialysis; ID, infectious diseases; ICU, intensive care unit; MRSA, methicillin-resistant *S. aureus;* MSSA, methicillin-susceptible *S. aureus;* SAB, *S. aureus* bacteremia.

## Data Availability

Not applicable.
